# Recent Advances in Pediatric Ventilatory Assistance

**DOI:** 10.12688/f1000research.10408.1

**Published:** 2017-03-17

**Authors:** Nicolas Nardi, Guillaume Mortamet, Laurence Ducharme-Crevier, Guillaume Emeriaud, Philippe Jouvet

**Affiliations:** 1 Pediatric Intensive Care Unit, CHU Sainte-Justine, University of Montreal, Montreal, Quebec, Canada

**Keywords:** ventilator-induced lung injury, diaphragmatic dysfunction, mechanical ventilation

## Abstract

In this review on respiratory assistance, we aim to discuss the following recent advances: the optimization and customization of mechanical ventilation, the use of high-frequency oscillatory ventilation, and the role of noninvasive ventilation. The prevention of ventilator-induced lung injury and diaphragmatic dysfunction is now a key aspect in the management of mechanical ventilation, since these complications may lead to higher mortality and prolonged length of stay in intensive care units. Different physiological measurements, such as esophageal pressure, electrical activity of the diaphragm, and volumetric capnography, may be useful objective tools to help guide ventilator assistance. Companies that design medical devices including ventilators and respiratory monitoring platforms play a key role in knowledge application. The creation of a ventilation consortium that includes companies, clinicians, researchers, and stakeholders could be a solution to promote much-needed device development and knowledge implementation.

## Introduction

Respiratory failure is the leading cause of admission to pediatric intensive care units (PICUs) ^1– 3^. Mechanical ventilation (MV) is a lifesaving therapy, allowing the support of patients with respiratory failure with the objectives of improving gas exchange and decreasing work of breathing. MV consists of a pressurized volume of gas delivered by either an invasive (tracheal tube or tracheostomy) or a non-invasive interface. MV is particularly challenging in children because of the heterogeneity of this population in terms of age, weight, and pathophysiology.

**Figure 1.**
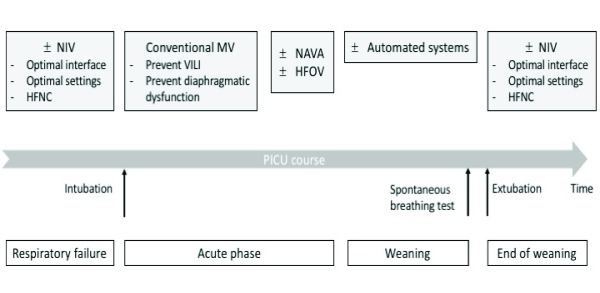
Schematic representation of recent advances in mechanical ventilation of critically ill children. HFNC, high-flow nasal cannula; HFOV, high-frequency oscillatory ventilation; MV, mechanical ventilation; NAVA, neurally adjusted ventilatory assist; NIV, noninvasive ventilation; VILI, ventilator-induced lung injury.

In this brief review, we aim to discuss the current clinical challenges in pediatric ventilatory assistance outside of the neonatal patient population. We will focus this discussion on recent advances regarding 1) optimization and individualization of patient–ventilator interactions during MV, 2) application of high-frequency oscillatory ventilation (HFOV), and 3) the role of noninvasive ventilation (NIV) ( Table 1 and Figure 1).

**Table 1.** Key messages suggested by the recent advances in pediatric ventilator assistance.

**Optimization/** **individualization of MV**	To limit ventilator-induced lung injury using transpulmonary pressure and volumetric capnography monitoring
	To limit diaphragmatic dysfunction by monitoring electrical activity of the diaphragm
	To better identify the timing of extubation with spontaneous breathing trials using CPAP mode or T-Tube
**Modes of MV**	To consider NAVA to improve patient–ventilator interaction
	To still consider high-frequency oscillatory ventilation in the most severe pediatric ARDS not adequately supported with optimally set conventional ventilation
**NIV**	To consider NIV as a first-line support in many pathologies
	To consider high-flow nasal cannula to improve comfort and tolerance of NIV
	To select the optimal interface according to the patient among all that are available nowadays

ARDS, acute respiratory distress syndrome; CPAP, continuous positive airway pressure; MV, mechanical ventilation; NAVA, neurally adjusted ventilatory assist; NIV, noninvasive ventilation.

## Advances in optimization and customization of mechanical ventilation in children

### Advances in the management of mechanical ventilation to limit ventilator-induced lung injury: transpulmonary pressure and capnography

The use of a global lung-protective ventilatory strategy, referring to low tidal volume and high levels of positive end-expiratory pressure (PEEP), in order to prevent ventilator-induced lung injury (VILI) improved survival in patients with acute respiratory distress syndrome (ARDS) ^4– 8^. In daily practice, the only way to assess the respiratory mechanics and the effects of MV on the lung itself are the ventilatory pressure, flow, and volume measured by the ventilator. However, relying on only these parameters during MV (plateau pressure level and tidal volume prescribed) may be misleading and may provide inaccurate assessment of the risk of VILI, since such variables do not accurately describe lung dynamics. Indeed, these recording variables reflect the respiratory system as a whole and do not take into account important pathophysiological features (e.g. chest wall compliance, intrinsic inspiratory/expiratory respiratory effort, heterogeneity of lung disease, etc.). Currently, new challenges are to optimize and customize MV by individualized monitoring at the bedside in order to avoid barotrauma, volotrauma, atelectrauma, and biotrauma ^9^. To do so, transpulmonary pressure and capnography monitoring are helpful.

The driving pressure is a key variable for clinicians to optimize protective volume and inspiratory pressure in order to avoid lung stress and strain ^5, 10^. The driving pressure is the ratio of the tidal volume to the static respiratory system compliance (ΔP=V _T_/C _RS_); it is also equivalent to the plateau pressure minus the PEEP (ΔP = Pplat – PEEP). A recent study by Amato *et al.* on adult ARDS reported that among different ventilation variables, driving pressure was most strongly and independently associated with survival. Indeed, a decrease in driving pressure concomitant to a reduction in tidal volume or an increase in PEEP were associated with increased survival, while differences in tidal volume were not associated with different survival rates when the driving pressure was constant ^5^. In ARDS, the proportion of lung available for ventilation is markedly decreased; therefore, the driving pressure (and consequently tidal volume) should be adapted to this reality rather than using only predicted body weight ^11^. However, it is important to note that this approach should be adapted depending on the patient’s condition. In particular, the impact of a given driving pressure might not be similar in patients with low chest wall compliance ^12^. It is the reason why there is an increasing interest in the monitoring of transpulmonary pressure to guide ventilatory assistance adjustments.

**Figure 2.**
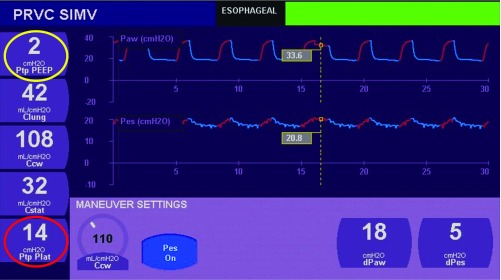
Esophageal and transpulmonary pressures measured in an 8-year-old morbidly obese patient with the Avea ventilator ^®^ from CareFusion. (1) Transpulmonary pressure (TPP = plateau pressure – esophageal pressure: to calculate TPP, esophageal pressure is used as a surrogate of intrapleural pressure) is 14 cmH _2_O at the end of insufflation (red circle). This means that despite a positive plateau pressure of 34 cmH _2_O in this obese child, lung parenchyma does not experience much distension (14 cmH _2_O) at the end of insufflation. (2) Positive end expiration pressure (PEEP) set on the ventilator is 2 cmH _2_O above esophageal pressure (yellow circle). This means that there is minimal risk of lung collapse at the end of expiration. Figure adapted from RM DiBlasi database with permission.

The transpulmonary pressure, defined by the difference between the airway pressure and pleural pressure, should be considered as the lung-distending pressure. This pressure measurement is closely correlated with lung strain and risk of VILI ^13^. Esophageal pressure is a good surrogate for pleural pressure and its measurement is valuable to the assessment of lung strain in mechanically ventilated patients. Despite controversies regarding the interpretation of absolute values of esophageal pressure, a recent paper reviewed the usefulness of this tool in ventilation management ^14^. Measurement of esophageal pressure is the only way to distinguish the effect of pressure on the lung and the chest wall. When a given amount of pressure is delivered, it is of great importance in some situations to better know which percentage is distending the lung (potentially harmful to the lungs) and which amount is distending the chest wall. As an example in ARDS, at the end of expiration, transpulmonary pressure can be negative (when pleural pressure exceeds end-expiratory airway pressure) and induces collapse of the alveoli. This may expose these parts of the lungs to being repeatedly reopened and recollapsed at each breath. To protect the lung from VILI, one would like to find a balance between protecting aerated units from over-distension and recruiting unstable units, thereby reducing tissue damage associated with their cyclic recruitment/derecruitment. The titration of PEEP based on esophageal pressure measurement ^15– 17^ has been proposed in patients with ARDS. Talmor *et al.* showed that oxygenation and lung compliance were significantly improved in patients managed by a ventilatory strategy including esophageal pressure measurement ^12^. This recent interest in transplumonary pressure has contributed to the development of such monitoring in several ventilators (Avea ventilator-CareFusion® and G5-Hamilton Medical®, for example). An example of clinical information given by esophageal pressure monitoring is given in Figure 2. Unfortunately, such ventilators are not available in all units while no dedicated monitor is able to provide this measurement. We believe that the use of transpulmonary pressure has to be developed and, nowadays, we include this monitoring in our clinical practice in the management of difficult-to-ventilate children with low lung and chest wall (and/or low abdominal) compliances. However, more research in this field is needed to validate the best strategy to quantify esophageal pressure in children and to confirm its utility in ventilation titration. In particular, the impact of mediastinum weight is taken into account by some authors in adult studies, but it has not been examined in pediatric patients. Beyond the estimation of absolute pleural pressure, we also use esophageal pressure monitoring to assess the work of breathing in invasive ventilation and NIV (see below) ^18– 20^.

Volumetric capnography (Vcap) is also a novel tool which allows the measurement of physiological and alveolar dead space at the bedside ^21– 23^. In this technique, expired CO _2_ is plotted against the tidal volume for each breath. Vcap analysis gives a global index of ventilation/perfusion (V/Q) mismatch, containing shunt and indices of lung efficiency (physiological and alveolar dead space). Vcap can help to set PEEP to obtain the lowest physiological and alveolar dead space, the lowest arterial to end-tidal CO _2_ gradient (PaCO _2_–ETCO _2_ gradient), and the optimal alveolar plateau slope (SIII) that reflect V/Q heterogeneity ^24– 27^. We believe that Vcap will help clinicians to set PEEP routinely in the near future. We already use it to better predict PaCO _2_ in mechanically ventilated children ^23^, and CO _2_ measurement obtained by Vcap is already included in a closed loop system dedicated to MV management ^28^.

### The role of diaphragmatic function in the management of mechanical ventilation

Increasing evidence suggests that MV is associated with diaphragmatic dysfunction and atrophy, also known as ventilator-induced diaphragmatic dysfunction ^29– 31^. To limit such consequences on the diaphragm, specific efforts should be addressed to reduce the duration of MV and to optimize ventilator settings. Improving individualized MV at bedside to limit diaphragmatic weakness is a great challenge but is essential to successfully wean patients from MV and decrease poor outcomes ^30, 32, 33^.

Monitoring of the electrical activity of the diaphragm (EAdi) provides new information to clinicians in order to assess diaphragm function and the impact of ventilation on the diaphragm muscle that can lead to rapidly progressive diaphragmatic weakness ^30, 32^. EAdi has been shown to reflect the patient ventilatory drive, and it is well correlated with work of breathing based on short-term physiological studies ^34, 35^. EAdi permits the detection of periods of blunted drive secondary to overassistance ^36^, which likely favor the risk of diaphragm dysfunction. It therefore may be used as a tool to adjust ventilatory support ^37^, to detect tonic activity of the diaphragm (which reflects the effort of the patient to increase the lung volume) ^38^, and to assess patient–ventilator asynchrony ^39^. When combined with pressure or volume delivered, EAdi measurements permit the assessment of diaphragm neuroventilatory (V _T_/EAdi) or neuromechanical (ΔP/EAdi) efficiency ^40^. In the only pediatric study on this topic to date, Wolf *et al*. observed that the ability to generate a higher diaphragmatic activity for the same tidal volume in pressure support ventilation (PSV) was a predictor of successful extubation ^41^.

**Figure 3.**
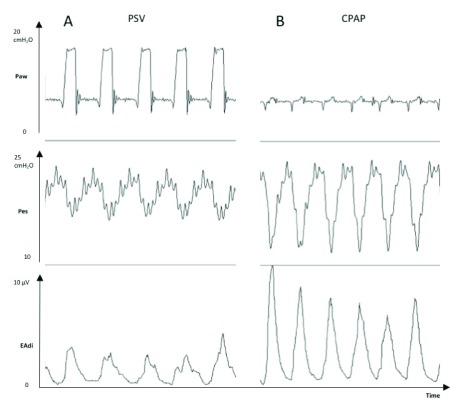
Tracings of a mechanically ventilated patient showing the increases in esophageal pressure swings (Pes, cmH _2_O) and electrical activity of the diaphragm (EAdi, μV) from a period with pressure support ventilation (PSV) +8 cmH _2_O and positive end-expiratory pressure +5 cmH _2_O (Panel **A**) to a period with continuous positive airway pressure (CPAP) +5 cmH _2_O only (Panel **B**). Paw, mean airway opening pressure.

As shown in Figure 3, both EAdi and esophageal pressure can provide similar clinical information regarding the patient’s work of breathing. Although these tools are correlated in most clinical situations, they can differ in patients with diaphragmatic dysfunction. It is therefore important to emphasize that EAdi represents respiratory drive and not diaphragmatic contractility.

This technology requires a specific nasogastric catheter equipped with distal electrodes (NAVA catheter, Maquet Critical Care, Solna, Sweden) connected to a dedicated Servo-I ventilator (Maquet Critical Care, Solna, Sweden). The main clinical application of EAdi monitoring is the neurally adjusted ventilatory assist mode (NAVA), a mode of ventilation which uses the EAdi to trigger and cycle-off breathing efforts. The NAVA level and the EAdi determine the amount of ventilator assistance. NAVA has many advantages compared to conventional MV, including improved patient–ventilator synchrony ^39, 42– 45^, the potential for a reduction in barotrauma (secondary to a decline of inspiratory pressure and tidal volume) ^23, 39, 42, 44, 46^, a possible decrease in atelectrauma ^47^, and, finally, improved diaphragmatic efficiency ^40^. Moreover, NAVA improves unloading of the respiratory muscles and prevents the risk of over-assistance through downregulation of EAdi induced by increased assistance ^37^. A recent randomized trial was conducted in children to test the clinical impact of NAVA ^48^. The feasibility of NAVA in clinical practice was confirmed, and NAVA was associated with lower FiO _2_ requirements and lower inspiratory pressures. A trend for shorter duration of ventilation was observed, but it did not reach statistical significance. Nowadays, we use the NAVA mode routinely, in particular in difficult-to-wean children, in children who have undergone cardiac surgery, or any case in which the promotion of assisted ventilation and avoidance of diaphragm rest is important. EAdi is also routinely used to detect diaphragm contractility recovery in children with neuromuscular disease (e.g. botulism, Guillain-Barré syndrome, and cervical trauma).

### Advances in weaning from mechanical ventilation

Owing to MV’s potential complications, such as VILI ^49^ and severe diaphragmatic atrophy ^30, 32^, it is imperative that it be discontinued as soon as the patient is capable of sustaining spontaneous breathing. On the other hand, premature extubation may also be problematic, as higher mortality rates have been reported in patients with extubation failure ^2, 50^. Consequently, when and how to perform MV weaning are key questions in critically ill patients. The identification of extubation readiness is usually based on clinical judgement, according to the respiratory, neurological, and hemodynamic status. However, this practice remains greatly subjective, while the timing of extubation is challenging. Therefore, efficient processes to safely reduce and remove ventilator support are necessary.

Clinical and research efforts have focused on early identification of weaning readiness. Some authors suggest the use of written protocols to assist clinicians in the management of weaning MV, but their usage in clinical practice remains limited for several reasons ^51^: (1) providing and following protocols are time consuming, resulting in fluctuation in protocol implementation and compliance; (2) clinical instructions may not be explicit enough, resulting in variable interpretations of the protocol; and (3) protocols are generally specific to one organization, leading to a certain heterogeneity in clinical practice.

The development of the closed-loop system (CLS) (computerized protocol implementing its recommendations without caregiver intervention) has resolved some of these issues ^52^. While optimizing ventilatory support on a continuous basis according to the patient’s respiratory condition, CLS offers consistent orders that constrain interpretation variations among caregivers, potentially resulting in a more efficient application of protocols. The use of CLS leads to a quicker adjustment of ventilator settings assessed by a reduction of time between the assessment of patient status and medical order, and medical order and clinical execution ^53^.

Two CLSs are commercialized for respiratory weaning: SmartCare/PS ^®^ (Dräger Medical, Lubeck, Germany) and IntelliVent ^®^ (Hamilton Medical, Bonaduz, Switzerland). These systems automatically reduce the level of support when the patient’s respiratory rate, tidal volume, and end tidal CO _2_ (EtPCO _2_) are within acceptable ranges. In adults, these systems reduced the weaning time without increasing adverse events ^54^. Currently, only two trials, one for each of these two technologies, have been conducted in children, and their findings regarding safety and duration of weaning process are encouraging ^28, 53^. A significant limitation of these systems remains the minimal weight/age required (15 kg for Smartcare/PS ^®^ and 7 kg with Intellivent ^®^) and they cannot be used in case of significant leaks around the endotracheal tube. We believe that these automated systems will improve the management of MV and therefore the outcome of patients, allowing the customization of ventilator support according to each child’s condition. However, companies and researchers should now focus their efforts on algorithms adapted to our pediatric population.

During the weaning process, identifying whether or not patients will be able to breathe spontaneously after extubation is a significant challenge. The recent consensus conference on pediatric ARDS (PALICC) has addressed this question and recommended that spontaneous breathing trials (SBTs) or extubation readiness tests should be performed ^55^.

Determining inclusion criteria for SBT initiation has been a difficult challenge because of the broad patient population, different modes of ventilation, and lack of consensus for acceptable SBT parameters. Another limitation is appropriate timing for starting SBT. For these reasons, some patients who qualify for SBTs may not be recognized, which may result in a prolonged ventilation course. Some institutions are now using electronic data pooled from ventilators and electronic medical records to develop explicit software rules and algorithms (decision support) to help identify patients who may be ready for SBT. Assuming a patient has met certain parameters for SBT criteria (EtCO _2_, SpO _2_, tidal volume, respiratory rate, inspiratory pressure, etc.), the electronic medical record can provide visual cues to help remind clinicians that their patient is ready for SBT.

In adults undergoing SBT, the use of an inspiratory pressure of 5 to 8 cmH _2_O is recommended ^56^. In children, very few data exist regarding the optimal method to conduct a SBT. Interestingly, a physiologic study conducted by Khemani *et al.,* comparing a SBT with a continuous positive airway pressure (CPAP) of 5 cmH _2_O versus pressure support of 10 cmH _2_O, concluded that pressure support significantly underestimates the potential for post extubation breathing efforts ^57^. According to this recent study, we recommend performing a SBT in CPAP mode or with a T-tube. However, it should be noted that respiratory efforts observed during CPAP trial will be reflective of the efforts observed after extubation but will be larger than during SBT with PSV. Therefore, it is not surprising to observe increased efforts during CPAP, which should not lead to delay in extubation unless they appear to be objectively poorly tolerated.

During weaning, esophageal pressure measurement can be a useful tool to assess the work of breathing. A robust parameter which can be derived from esophageal pressure and transdiaphragmatic pressure, i.e. the difference between esophageal pressure and gastric pressure, is the pressure-time-product. This parameter was used as a tool to assess work of breathing and optimize ventilation support in children with different diseases ^18, 20^. Jubran *et al.* showed that esophageal pressure trend during a SBT provided an accurate prediction of weaning outcome ^58^. Over the course of a SBT, esophageal pressure-time-product remained unchanged in successfully weaned patients. In contrast, weaning failure patients developed marked and progressive increase in esophageal pressure-time-product (up to 4-fold above the normal value) as a result of an increase in the mechanical load of the respiratory muscles ^58^.

## Advances in high-frequency oscillatory ventilation

HFOV has been commonly used for decades in neonatal, pediatric, and adult populations ^58^. Clinical trials have demonstrated that HFOV is associated with an oxygenation improvement in patients with acute lung injury or ARDS ^59– 61^. However, the clinical use of HFOV in this population has decreased. Recent studies demonstrated an association between early use of HFOV and worse outcome in terms of mortality in adult ^62^ and pediatric populations ^63, 64^. However, several biases have been highlighted in the two pediatric studies regarding the methodology ^65– 67^. As suggested by Rettig *et al.*, the mortality in patients with ARDS supported by HFOV may be linked to the disease category itself rather than the use of HFOV ^68^. Given these limitations and with regard to our clinical experience, we consider, as supported by the PALICC, HFOV to still be a rescue therapy in some children with severe ARDS.

## Advances in noninvasive ventilation

NIV is defined as the delivery of MV without an endotracheal tube or tracheostomy. NIV comprises both CPAP and bilevel positive airway pressure (BiPAP) ventilation. NIV is increasingly used in PICUs ^69, 70^. In the last decade, the potential indications for NIV in critically ill patients have grown considerably, and the performance of this mode of support has greatly improved. In children developing ARDS, NIV can be considered as a first line of treatment in milder disease ^55^. Despite the lack of clear guidelines, this mode of support definitely has its place in the treatment of a wide range of pathologies in children, including pneumonia, upper airway obstruction, post-extubation respiratory failure, acute chest syndrome, and asthma ^70^.

The use of NIV has recently evolved because of the emergence of high-flow nasal cannula (HFNC). This modality is now available from a number of manufacturers and has been widely adopted in pediatric practice. Different mechanisms have been hypothesized to account for the clinical benefits, including washout of the nasopharyngeal dead space, reduction of work of breathing, decrease in airway resistance, and improvement of pulmonary compliance ^71, 72^. HFNC has been able to provide a mean pharyngeal pressure of 4 cmH _2_O when used at a flow of 2 L/kg/minute ^73^, but this effect is variable. In clinical use, HFNC allows improvement of comfort and tolerance to NIV and reduction of air leak, gastric distension, and skin injuries, especially in younger children. The literature is still poor to identify the specific population that would benefit from this technology ^18, 74^. The role of HFNC outside the PICU still needs to be investigated, and we currently restrict HFNC use in the PICU. More evidence is expected from several ongoing randomized controlled trials (TRAMONTANE study, NCT02457013; Hi-Flo study, NCT01498094; HHFNC study, NCT01662544). We believe that, within a few years, the role of HFNC will be better defined and potentially widened.

The optimal interface for NIV in children has recently been discussed as a key aspect in respiratory management ^75^. A large variety of devices recently emerged, including nasal, oronasal, and total face masks and helmet. Because mask-fit pressure is spread over a larger surface beyond the nose area, total face masks appear to be more comfortable than oronasal masks ^76^. This device was shown to be as efficient as oronasal mask in terms of breathing pattern, gas exchange, and outcome in adults ^77^. The helmet is also increasingly used ^70^ and should be considered as a feasible alternative for NIV in children, as suggested by the results of a recent randomized controlled trial comparing the use of a helmet and a face mask in children ^78^. As for total face masks, preliminary data are pointing towards the helmet as an interface to increase comfort and decrease skin injury and air leaks ^79^.

Finally, to improve NIV success, the achievement of an adequate patient–ventilator synchrony is crucial ^19^. Although the performance of ventilators has improved within the last few years, patient–ventilator asynchrony in NIV remains a significant issue. As with invasive ventilation, tools to improve patient–ventilator synchrony during NIV have been recently investigated. EAdi monitoring and noninvasive NAVA are feasible and well tolerated in PICU patients with patient–ventilator synchrony improvement ^80, 81^. Monitoring esogastric pressure offers another way to improve patient–ventilator interaction during NIV. In infants ^82^ and children ^19^, esophageal pressure measurement has been shown to be a valuable tool to assess patient–ventilator interaction and to optimize ventilatory settings ( Figure 1).

## Conclusion

There have been major advances in the management of mechanically ventilating children over the last 3 years. The implementation of this new knowledge in usual practice is a challenge, as advances occur not only in the respiratory field but also in many fields that pediatric intensivists must digest. In such a situation, companies that design medical devices including ventilators and respiratory monitoring platforms play a key role in the application of knowledge. The creation of a ventilation consortium that includes companies, caregivers, researchers, and stakeholders could be a solution to promote knowledge implementation.
